# White Matter Connectivity and Gray Matter Volume Changes Following Donepezil Treatment in Patients With Mild Cognitive Impairment: A Preliminary Study Using Probabilistic Tractography

**DOI:** 10.3389/fnagi.2020.604940

**Published:** 2021-03-16

**Authors:** Gwang-Won Kim, Shin-Eui Park, Kwangsung Park, Gwang-Woo Jeong

**Affiliations:** ^1^Advanced Institute of Aging Science, Chonnam National University, Gwangju, South Korea; ^2^Department of Psychiatry, Massachusetts General Hospital and Harvard Medical School, Boston, MA, United States; ^3^Department of Urology, Chonnam National University Hospital, Chonnam National University Medical School, Gwangju, South Korea; ^4^Department of Radiology, Chonnam National University Hospital, Chonnam National University Medical School, Gwangju, South Korea

**Keywords:** white matter connectivity, probabilistic tractography algorithm, mild cognitive impairment, gray matter volume, donepezil treatment, cortical thickness

## Abstract

The donepezil treatment is associated with improved cognitive performance in patients with mild cognitive impairment (MCI), and its clinical effectiveness is well-known. However, the impact of the donepezil treatment on the enhanced white matter connectivity in MCI is still unclear. The purpose of this study was to evaluate the thalamo-cortical white matter (WM) connectivity and cortical thickness and gray matter (GM) volume changes in the cortical regions following donepezil treatment in patients with MCI using probabilistic tractography and voxel-based morphometry. Patients with MCI underwent magnetic resonance examinations before and after 6-month donepezil treatment. Compared with healthy controls, patients with MCI showed decreased WM connectivity of the thalamo-lateral prefrontal cortex, as well as reduced thickness in the medial/lateral orbitofrontal cortices (*p* < 0.05). The thalamo-lateral temporal cortex connectivity in patients with MCI was negatively correlated with Alzheimer's disease assessment scale-cognitive subscale (ADAS-cog) (*r* = −0.76, *p* = 0.01). The average score of the Korean version of the mini-mental state examination (K-MMSE) in patients with MCI was improved by 7.9% after 6-months of donepezil treatment. However, the patterns of WM connectivity and brain volume change in untreated and treated patients were not significantly different from each other, resulting from multiple comparison corrections. These findings will be valuable in understanding the neurophysiopathological mechanism on MCI as a prodromal phase of Alzheimer's disease in connection with brain functional connectivity and morphometric change.

## Introduction

Alzheimer's disease (AD) is a chronic brain disorder that is associated with neurodegeneration and the progressive development of dementia (Fumagalli et al., 2006). AD generally progresses slowly in three stages, including preclinical AD, mild cognitive impairment (MCI) due to AD pathology, and AD-dementia. MCI is a prodromal stage of AD, which is a relatively broad clinical condition that involves a slight memory deficit, and in many cases, the condition may represent a transitional state between normal cognition and AD (Morris, 2005). Among the neuropathological alterations in AD, relevant neuronal loss, and synaptic pathology have been studied to be most strongly related to dementia severity and cognitive deficits (Hoy et al., 2017). White matter (WM) degeneration occurs early in the development of AD and is useful in evaluating pathologic AD progression before AD becomes clinically evident (Caso et al., 2015). Several postmortem studies have reported that the early neuropathology in AD manifests in the medial temporal lobe (Hyman et al., 1984; Krasuski et al., 1998).

The key clinical symptom of AD is the progressive deterioration of learning and memory, which further leads to reduced acetylcholine (ACh) levels in the brain (Hashimoto et al., 2005). Treatment with acetylcholinesterase inhibitors (AChEIs) prevents the breakdown of ACh and impacts an increase in cholinergic transmission. Acetylcholinesterase inhibitors are among the approved drugs currently used to treat AD, wherein, and the most frequently prescribed drug is donepezil (Cavedo et al., 2017). Donepezil has been demonstrated to inhibit acetylcholinesterase activity in the cerebral cortex, hippocampus, and striatum of the rat brain, by impacting the increased production of ACh activity in the brain areas associated with cognitive function (Kasa et al., 2000; Scali et al., 2002). Kim et al. (2020) reported that donepezil treatment in patients with MCI is associated with improved cognitive performance. However, it is as yet unknown how donepezil treatment influences the brain cortical thickness and WM connectivity in MCI.

Diffusion weighted imaging (DWI) facilitates measuring the effects of tissue microstructure on the random translational motion of water molecules in biologic tissues and has been reported to be highly sensitive to WM microstructural damage (Caso et al., 2015). Several DWI studies (Wang et al., 2012; Mito et al., 2018) focusing on MCI and AD have analyzed the DWI data based on the diffusion tensor model. For the detection of WM integrity of an entire bundle, probabilistic tractography in DTI has recently emerged as an increasing medium, allowing us to evaluate structural connectivity through estimating the likelihood that two areas of the brain are interconnected (Jaimes et al., 2017). This tool may advantageously provide more valuable information and insight regarding the early signs of microstructural change in MCI and brain structural connectivity following the donepezil treatment. Most brain structural connectivity studies focusing on MCI and AD have exclusively explored the specific brain region-of-interest (ROI), hippocampus, or medial temporal lobe. AD has largely been considered a disease of the cerebral cortex, and thus it is important to screen cortical dysfunction at an early stage before the development of AD. The thalamus is an evolutionarily conserved structure with extensive reciprocal connections to cortical regions, and it plays an important role in learning and memory (Nakajima and Halassa, 2017). Alderson et al. (2017) have reported that patients with MCI showed significantly decreased fractional anisotropy between the thalamus and inferior parietal lobe compared with healthy controls. To date, however, no neuroimaging studies on the interaction between the thalamus and cortical regions using probabilistic tractography in patients with MCI following donepezil treatment have been attempted. We have previously reported on a study that evaluated the thalamo-cortical WM connectivity in patients with MCI using probabilistic tractography. Voxel-based morphometry (VBM) has grown in popularity since its introduction because of its ability to perform statistical tests across all voxels in the image, identifying volume differences between groups (Whitwell, 2009; Kim et al., 2018b). Therefore, a combined study of probabilistic tractography and VBM could further understanding of gray matter (GM) atrophy and the WM networks that can cause disconnection among neural cells in MCI.

The purpose of this study was to evaluate the thalamo-cortical WM connectivity before and after donepezil treatment in patients with MCI using probabilistic tractography, as well as to assess the cortical thickness and GM volume changes in the cortical regions.

## Subjects and Method

### Ethics

This is study a retrospective that has been approved by the Institutional Review Board of Chonnam National University Hospital (IRB-CNUH). Before MR scanning, the experimental procedure was explained to all volunteers, and written informed consent was obtained. All the experimental procedures and methods were performed in accordance with the relevant guidelines and regulations approved by IRB-CNUH.

### Subjects

Ten patients with MCI (male:female = 4:6, mean age = 72.4 ± 7.9 years) and 9 age-matched healthy controls (male:female = 3:6, mean age = 70.7 ± 3.5 years) participated in this study (Table 1). The patients with MCI were inpatients or outpatients of the CNUH. We included the patients with MCI based on the following criteria: first, the MCI of Alzheimer-type by the criteria of both the DSM-IV and the National Institute of Neurological and Communicative Diseases and Stroke-Alzheimer Disease and Related Disorders Association (NINCDS-ADRDA); second, no history of MCI treatment and other neurological or psychiatric illnesses; third, a score of 0.5 or 1 on the Clinical Dementia Rating (CDR); fourth, a score <26 on the Korean version of the Mini-Mental State Examination (K-MMSE); fifth, reconfirmation through the typical symptom severity including change in cognition recognized by the affected individual or observers, objective impairment in one or more cognitive domains, independence in functional activities, and absence of dementia (Morris, 2012).

**Table 1:** Demographic and clinical characteristics of patients with MCI (baseline), donepezil-treated patients (follow-up), and healthy controls (HC).

	**MCI patients**		**Healthy controls** **(n = 9)**	**Statistical analysis** **(*****p*****-value)**		
	**Baseline** **(*n* = 10)**	**Follow-up** **(*n* = 10)**		**Baseline vs.** **follow-up**	**HC vs.** **baseline**	**HC vs.** **follow-up**
Age (years)	73.1 ± 7.9	-	70.7 ± 3 .5	-	*p* = 0.092	*p* = 0.092
Gender	7 F, 3 M	-	6 F, 3 M	-	*p* = 0.876	*p* = 0.876
K-MMSE	16.5 ± 4.9	17.5 ± 2.9	28.6 ± 1.1	*p* = 0.031^a^	*p* < 0.001^b^	*p* < 0.001^b^
ADAS-Cog	25.6 ± 6.2	24.4 ± 5.9	-	*p* = 0.506	-	-
CDR	0.6 ± 0.2	0.6 ± 0.2	-	*p* = 0.317	-	-
GDS	13.2 ± 5.2	12.7 ± 4.9	-	*p* = 0.372	-	-

a Significant difference (Wilcoxon's signed-ranks; p < 0.05) between MCI patients (baseline) and treated patients (follow-up).

b Significant differences (Mann-Whitney U; p < 0.001) in both “healthy controls (HC) vs. MCI patients” and “HC vs. treated patients”.

*K-MMSE, the Korean version of the mini-mental state examination; ADAS-Cog, AD assessment scale-cognitive subscale; CDR, clinical dementia rating; GDS, geriatric depression scale*.

Ten patients underwent MR examinations before (baseline) and after (follow-up) donepezil treatment. After performing the first MR examination, 10 patients received 5 mg/day of Aricept (donepezil hydrochloride; Pfizer Inc., New York, NY) for the initial 28 days, and 10 mg/day thereafter. The mean time gap between before and after donepezil treatment was 194.0 ± 29.5 days.

### Neuropsychological Tests

The MCI symptom severity was evaluated in the two separated groups, receiving and not receiving donepezil treatment, using the questionnaires of the K-MMSE, AD assessment scale-cognitive subscale (ADAS-Cog), CDR scale, and geriatric depression scale (GDS). Patients with MCI completed questionnaires before and after the 6-months of donepezil treatment. The contrasts of “healthy controls vs. patients with MCI” and “healthy controls vs. using donepezil-treated patients” were analyzed by using a Mann-Whitney *U*-test. A Wilcoxon's signed-ranks test was used to compare the scores on the K-MMSE, ADAS-Cog, CDR, and GDS between untreated and treated patients.

### Image Acquisition

T1- and T2-weighted images and DWI were performed on a 3.0-T Magnetom Tim Trio MR Scanner (Siemens Medical Solutions, Erlangen, Germany) with a head coil of birdcage type. The axial DW images were acquired using echo-planar imaging pulse sequence with the following parameters: TR/TE = 5,200/105 ms, matrix = 128 × 128, field of view (FOV) = 220 mm^2^, and resolution = 1.7 mm × 1.7 mm. Diffusion sensitizing gradient-echo encoding was applied in 24 directions using a diffusion-weighting b factor of 1,000 s/mm^2^ and 5 images without diffusion weighting (b factor = 0 s/mm^2^). Phase-encoding was in the anterior → posterior direction and a factor of 2 in-plane acceleration (GRAPPA) was used. T1-weighted sagittal images (TR/TE = 1,700/2.2 ms) and T2-weighted axial images (TR/TE = 5,000/90 ms) were acquired with the following parameters: FOV = 256 × 256 mm^2^, matrix = 512 × 512, slice thickness = 5 mm, and slice gap = 2 mm.

### Data Processing and Analysis

**Figure 1:**
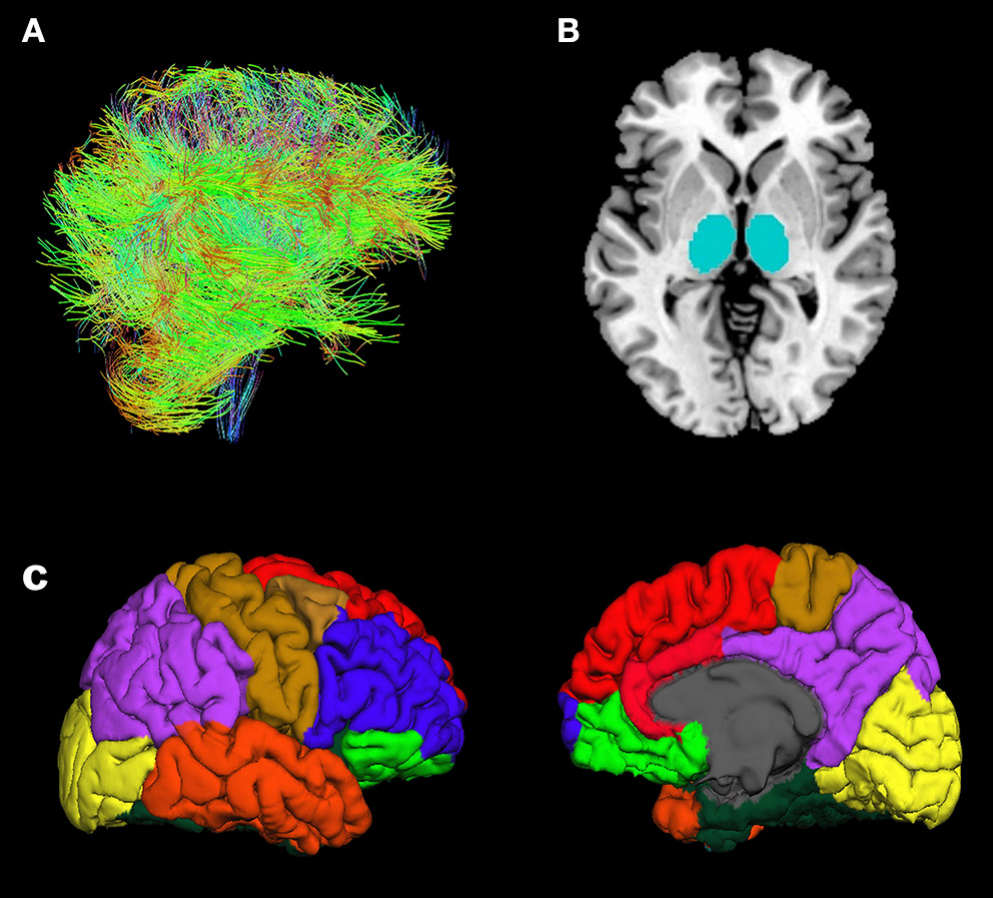
Illustration of fiber tracts **(A)**, thalamus ROIs (seed regions) **(B)**, and cortical ROIs (target regions) **(C)**: orbitofrontal cortex (yellow-green), medial prefrontal cortex (red), lateral prefrontal cortex (blue), sensorimotor cortex (brown), parietal cortex (purple), lateral temporal cortex (orange), medial temporal cortex (dark-green), and occipital cortex (yellow).

DWI data were analyzed using Functional Magnetic Resonance Imaging of the Brain (FMRIB) Software Library (FSL) v6.0 software (Behrens et al., 2003, 2007; Jenkinson et al., 2012). Based on the findings of previous studies (Marenco et al., 2012; Cho et al., 2016) that used probabilistic tractography, we identified 9 brain regions of interest (ROIs) in all, individual T1 images using the FreeSurfer v6.0 software (MGH, Boston, MA, USA) with Desikan-Killiany cortical parcellation as following: seed region; thalamus, target regions; orbitofrontal cortex (OFC), medial prefrontal cortex (MPFC), lateral prefrontal cortex (LPFC), sensorimotor cortex (SMC), parietal cortex (PC), medial temporal cortex (MTC), lateral temporal cortex (LTC), and occipital cortex (OC) (Figure 1) (Dale et al., 1999; Fischl et al., 2002, 2004). The DWI data were preprocessed using skull removal and eddy current as well as motion correction. The first non-diffusion weighted image was set as the target image, into which the remaining images (24 diffusion weighted image and 4 non-diffusion weighted images) were registered using an affine transformation to adjust for distortions caused by eddy currents and head motion (Tsai, 2018). The individual T1 images were rigidly registered to their corresponding non-diffusion weighted (B0) images using FMRIB's Linear Image Registration Tool (FLIRT) in combination with mutual information cost function and trilinear interpolation. Diffusion parameters were modeled using Bayesian Estimation of Diffusion Parameters Obtained using Sampling Techniques (BEDPOSTX) with crossing-fibers modeling. One patient had motion artifact in the T1 images obtained after treatment, thus nine ROIs in the patient were extracted in the T1 image obtained before treatment to register their T1 images to diffusion space. BEDPOSTX models of diffusion signal as a ball (isotropic) and stick (anisotropic) components were used to generate a distribution of likely fiber orientations within each voxel as well as an estimate of the uncertainty on these orientations (Theisen et al., 2017). To determine WM connectivity between seed region and target regions, we used FSL probabilistic tractography (connectivity modeling) as following: 5,000 streamlines per each voxel in the thalamus, 0.2 curvature threshold, 0.5 mm step length, and loop check. The connectivity values were routinely thresholded at 10% to remove the aberrant connections arising from noise and errors (Cho et al., 2016). To take into account the individual variances in total connectivity between the thalamus and eight cortical ROIs, each thalamo-cortical WM connectivity value was divided by the sum of connectivity values from all cortical ROIs, named “relative connectivity” (Cho et al., 2016).

For the group analysis, a Wilcoxon's signed-ranks test was conducted using the SPSS (version 24.0, IBM, Armonk, NY, USA) to compare the thalamo-cortical WM connectivity between patients receiving and not receiving donepezil treatment. A Mann-Whitney U-test was used to compare the connectivity between patients and healthy controls. The significance level was set to 0.05 after Bonferroni correction for 8 brain cortices to adjust multiple comparisons (level of significance after Bonferroni correction: *p* < 0.0063).

The cortical thickness of the entire brain was calculated using the FreeSurfer v6.0 software (MGH, Boston, MA, USA). T1 data of 1 subject was excluded from the cortical thickness and VBM analyses due to a motion artifact detected in the anatomical scan. Post-processing of images comprised the following steps: correction for head motion and non-uniformity of intensity, Talairach transformation of each subject's brain, removal of non-brain tissue, segmentation of cortical gray, subcortical white and deep GM volumetric structures, triangular tessellation of the GM/WM matter interface and GM/CSF boundary, and topology correction. The images were then smoothed with a 10-mm FWHM Gaussian kernel. Cortical thickness was calculated as the shortest distance between the GM/WM boundary and pial surface at each vertex across the cortical mantle, measured in millimeters (Gerrits et al., 2016). The cortical maps were generated by computing mean cortical thickness for each subject at each vertex, right and left hemispheres separately, and mapping these data to the surface of an average brain template enabling visualization of data across the entire cortical surface (Han et al., 2014). Cortical thickness was compared between patients receiving and not receiving donepezil treatment using Wilcoxon's signed-ranks test, and between patients and healthy controls using the Mann-Whitney U-test.

Brain GM volume was analyzed using SPM8 software (Statistical Parametric Mapping, Wellcome Department of Cognitive Neurology, University College, London, U.K.) with diffeomorphic anatomical registration through exponentiated Lie algebra (DARTEL) analysis (Kim et al., 2018a, 2019). Prior to processing data, all individual T1 images were aligned with the anterior and posterior commissure line on the transverse plane. After correcting the non-uniformity field bias on images, the images were segmented to GM, WM, and cerebrospinal fluid (CSF) using the tissue probability maps based on the International Consortium of Brain Mapping (ICBM) space template type of East Asian Brains. In addition, the mean templates of GM and WM were created using individual GM and WM images. Subsequently, all the images were normalized to the Montreal Neurological Institute template and smoothed with an 8 mm full width at a half maximum (FWHM) isotropic Gaussian kernel.

To compare the GM volumes between healthy controls and patients with MCI before or after donepezil treatment, a two sample *t*-test, with whole brain volume as covariate, was used in the Statistical non-Parametric Mapping (SnPM13). A paired *t*-test was used to compare the GM volumes between patients with MCI and treated patients with MCI. The results were thresholded at a cluster level corrected threshold of *p* < 0.05 [*n* = 5,000 permutations, family-wise error (FWE)-corrected] with a cluster-determining threshold at the voxel level *p* < 0.0001.

## Results

### Changes in Symptom Severity

The average scores of K-MMSE in healthy control, patients with MCI, and donepezil-treated patients with MCI were 28.6 ± 1.1, 16.5 ± 4.9, and 17.5 ± 2.9, respectively (Table 1). The average score of K-MMSE in untreated patients with MCI was improved by 7.9% after donepezil treatment (*p* = 0.031). The average scores of ADAS-Cog and GDS in untreated patients were decreased by 5.0% (25.6 ± 6.2 → 24.4 ± 5.9; *p* = 0.506) and 4.0% (13.2 ± 5.2 → 12.7 ± 4.9; *p* = 0.372) after donepezil treatment, respectively (Table 1). In addition, the average score of CDR in untreated patients and donepezil-treated patients were 0.6 ± 0.2, and 0.6 ± 0.2, respectively (*p* = 0.317) (Table 1).

### White Matter Connectivity Changes

**Figure 2:**
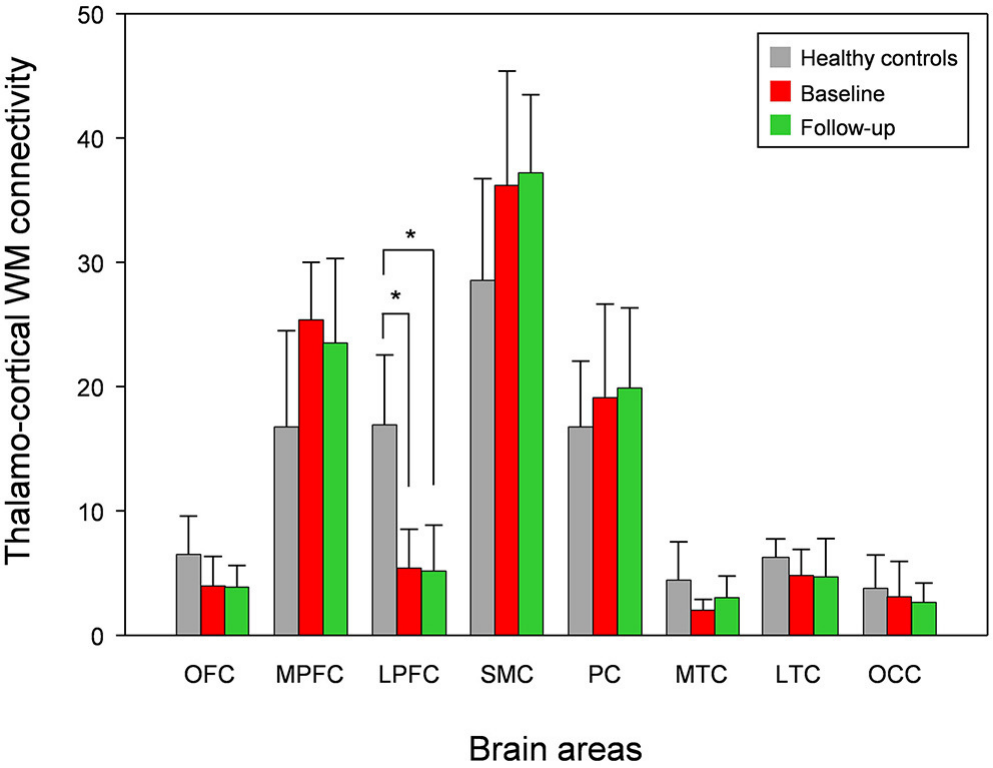
Mean white matter (WM) connectivity between the thalamus and each cortex in patients with MCI (baseline), donepezil-treated patients (Follow-up), and healthy controls. OFC, orbitofrontal cortex; MPFC, medial prefrontal cortex; LPFC, lateral prefrontal cortex; SMC, sensorimotor cortex; PC, parietal cortex; MTC, medial temporal cortex; LTC, lateral temporal cortex; OC, occipital cortex. *significant difference (Bonferroni corrected, *p* < 0.05).

**Figure 3:**
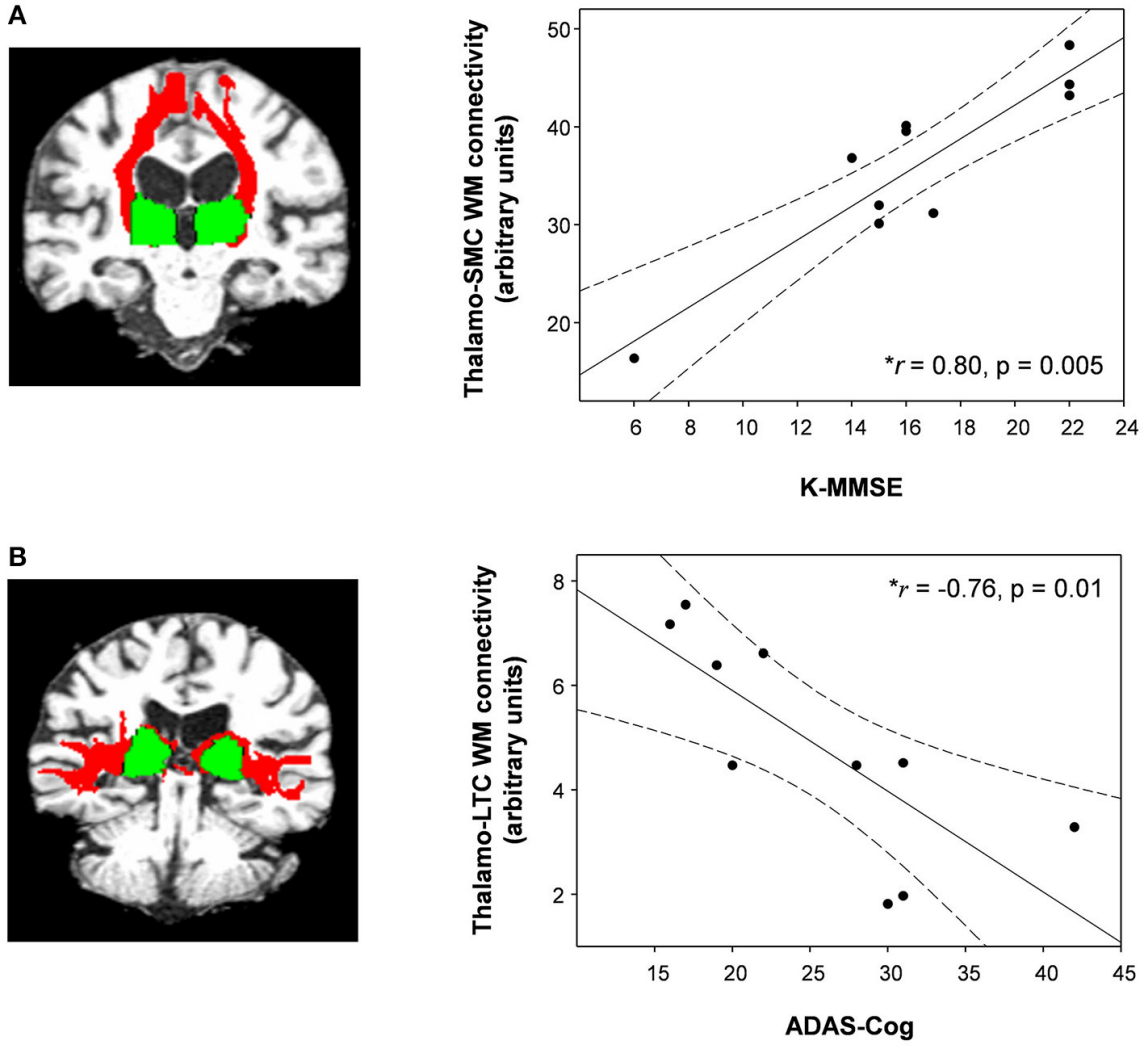
**(A)** The thalamo-sensorimotor cortex (SMC) white matter connectivity in patients with MCI was positively correlated with K-MMSE scores (Spearman's rho = 0.80, *p* = 0.005) and **(B)** the thalamo-lateral temporal cortex (LTC) was negatively correlated with ADAS-cog scores (Spearman's rho = −0.76, *p* = 0.01). Dotted lines show 95% confidence intervals. Red, white matter connectivity; green, thalamus ROIs (seed regions). • Shows WM connectivity and K-MMSE or ADAS-Cog in patients with MCI.

Compared with healthy controls, the untreated and treated patients showed significantly decreased thalamo-LPFC relative connectivity (*p* < 0.05, Bonferroni corrected) (Figure 2, Table 2). Treated patients showed increased thalamo-MTC relative connectivity after donepezil treatment (*p* < 0.05). Interestingly, thalamo-LPFC relative connectivity in patients with MCI was positively correlated with the K-MMSE (*r* = 0.80, *p* = 0.005) and thalamo-LTC relative connectivity was negatively correlated with the ADAS-Cog (*r* = −0.76, *p* = 0.010) (Figure 3).

**Table 2:** Mean white matter connectivity between the thalamus and each cortex in patients with MCI (baseline), donepezil-treated patients (follow-up), and healthy controls (HC).

**Brain cortex**	**MCI patients (baseline)**	**Treated patients (follow-up)**	**Healthy controls (HC)**	**Statistical analysis**		
				**Baseline vs. follow-up**	**HC vs. baseline**	**HC vs. follow-up**
OFC	0.04 ± 0.02	0.04 ± 0.02	0.06 ± 0.03	*p* = 0.959	*p* = 0.027	*p* = 0.034
MPFC	0.25 ± 0.05	0.24 ± 0.07	0.17 ± 0.08	*p* = 0.575	*p* = 0.014	*p* = 0.086
LPFC	0.05 ± 0.03	0.05 ± 0.04	0.17 ± 0.06	*p* = 0.959	^*^p < 0.001	^*^p = 0.001
SMC	0.36 ± 0.09	0.37 ± 0.06	0.29 ± 0.08	*p* = 0.575	*p* = 0.060	*p* = 0.022
PC	0.19 ± 0.08	0.20 ± 0.06	0.17 ± 0.05	*p* = 0.959	*p* = 0.514	*p* = 0.221
MTC	0.02 ± 0.01	0.03 ± 0.02	0.04 ± 0.03	*p* = 0.037	*p* = 0.022	*p* = 0.221
LTC	0.05 ± 0.02	0.05 ± 0.03	0.06 ± 0.01	*p* = 0.444	*p* = 0.165	*p* = 0.050
OCC	0.03 ± 0.03	0.03 ± 0.02	0.04 ± 0.03	*p* = 0.445	*p* = 0.327	*p* = 0.253

OFC, orbitofrontal cortex; MPFC, medial prefrontal cortex; LPFC, lateral prefrontal cortex; SMC, sensorimotor cortex; PC, parietal cortex; MTC, medial temporal cortex; LTC, lateral temporal cortex; OC, occipital cortex.

* *significant difference (Bonferroni corrected, p < 0.05)*.

### Cortical Thickness

**Figure 4:**
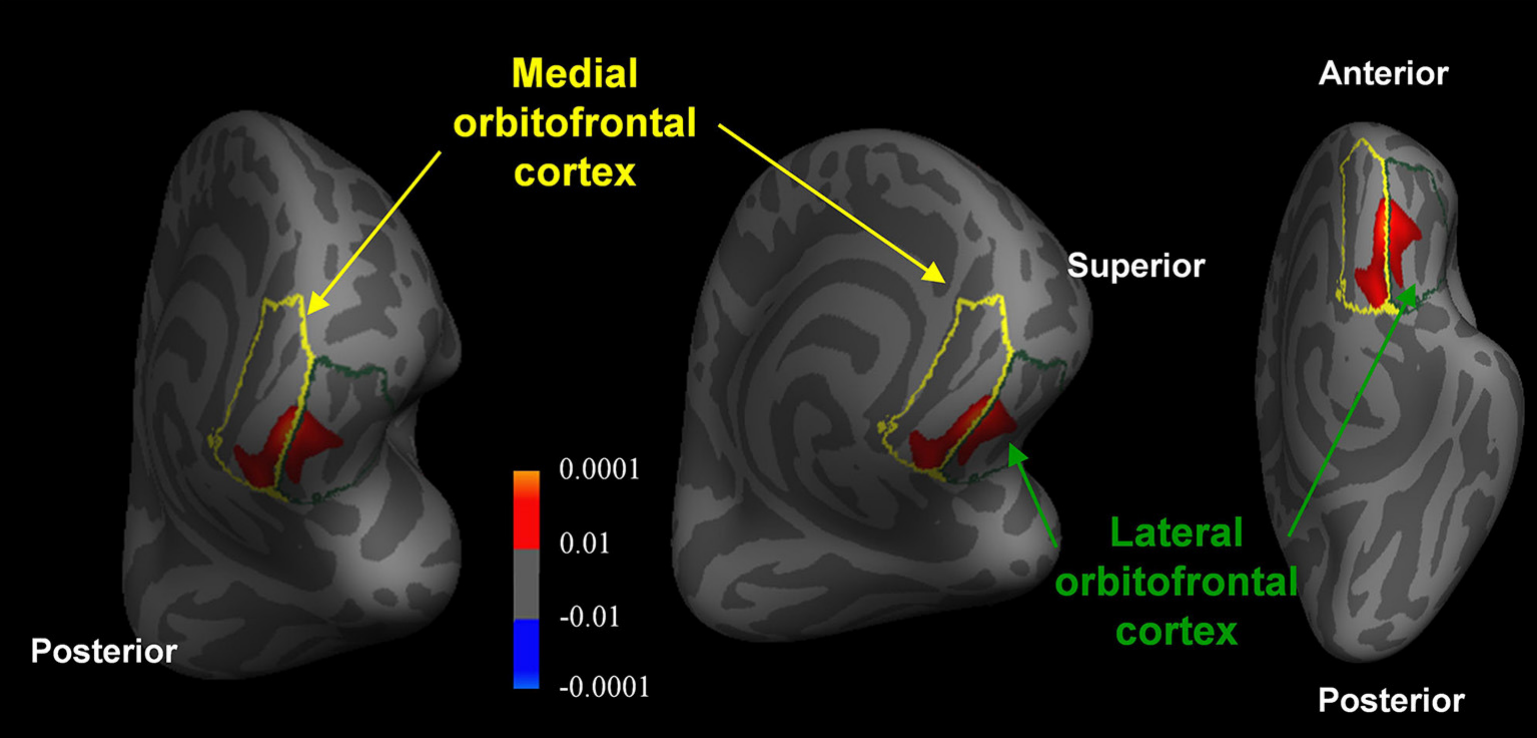
Reduced orbitofrontal cortex thickness in patients with MCI compared with healthy controls (Monte-Carlo corrected, *p* < 0.01). The *p*-values of the clusters are represented by warm (healthy controls > MCI patients) and cool (MCI patients > healthy controls) colors.

Compared with the healthy controls, patients with MCI showed significantly reduced thickness in the medial orbitofrontal cortex ([x, y, z = −9, 37, −21], *t*-value = 3.6) and lateral orbitofrontal cortex ([x, y, z = −13, 34, −19], *t*-value = 3.0) (Monte-Carlo corrected, *p* < 0.01) (Figure 4). However, no significant differences were found between untreated and treated patients.

### Global Gray Matter Volume Changes

**Figure 5:**
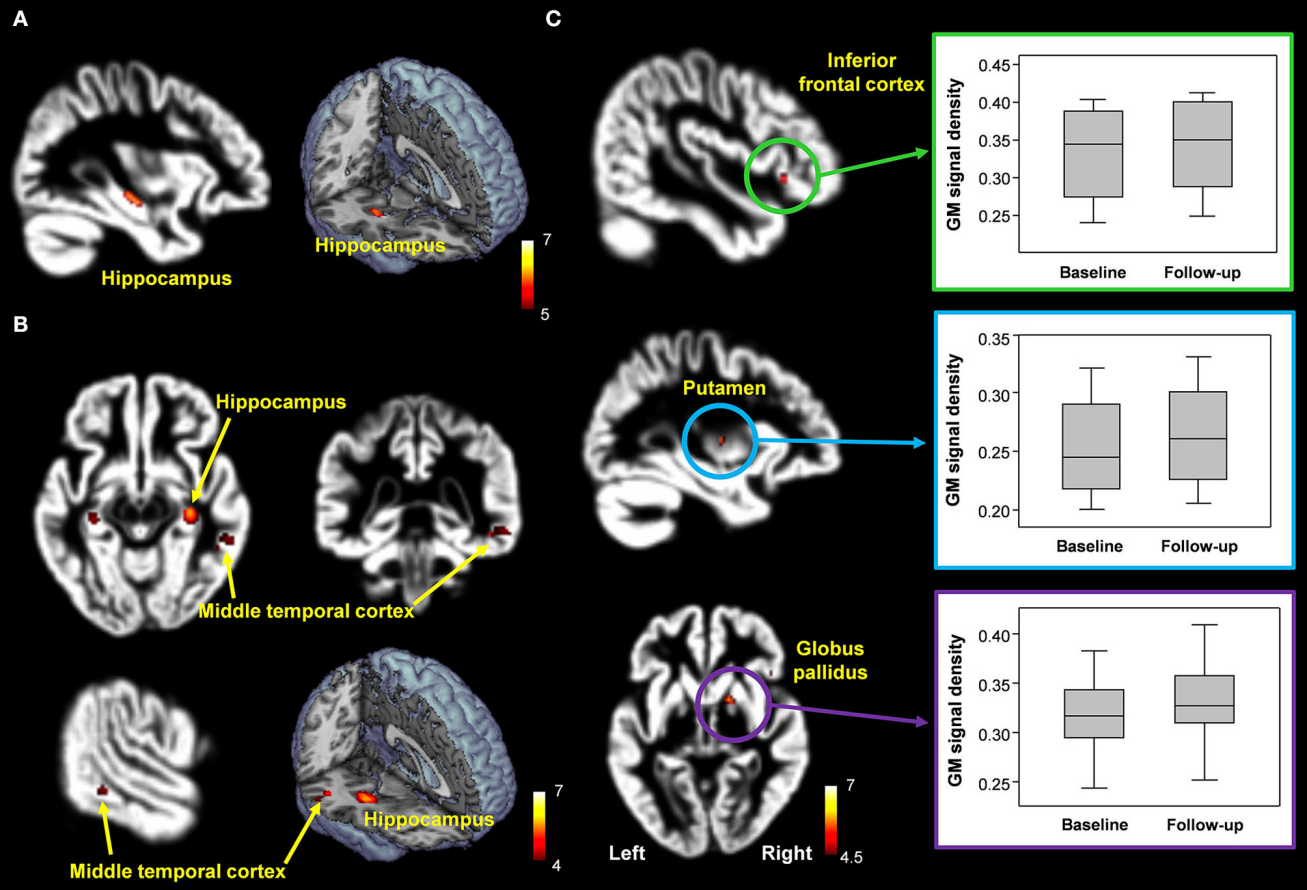
**(A,B)** Decreased hippocampal volume in patients with MCI compared with healthy controls [FWE corrected, *p* < 0.05 **(A)**; uncorrected *p* < 0.0005 **(B)**]. **(C)** Brain regions with significantly increased GM volumes in donepezil-treated patients compared with patients with MCI (uncorrected, *p* < 0.001).

The overall GM volumes in healthy control, patients with MCI, and donepezil-treated patients were 589.9 ± 54.7 mL, 574.0 ± 36.8 mL, and 578.9 ± 32.8 mL, respectively. In comparison with the healthy controls, patients with MCI showed significantly reduced GM volumes in the hippocampus ([x, y, z = 32, −16, −14], *t*-value = 5.8) (FWE corrected, *p* < 0.05) (Figure 5). Furthermore, when comparing the untreated and treated patients, the treated patients showed significantly higher GM volumes in the putamen ([x, y, z = −30, −10, 6], *t*-value = 5.5), globus pailldus ([x, y, z = 16, 6, 0], *t*-value = 6.0), and inferior frontal cortex ([x, y, z = 46, 24, −4], *t*-value = 5.2) (uncorrected; *p* < 0.001) (Figure 5).

## Discussion

To our knowledge, this is the first study evaluating the thalamo-cortical WM connectivity after donepezil treatment in patients with MCI using probabilistic tractography. Patients with MCI showed decreased thalamo-LPFC WM connectivity, as well as reduced thickness in the medial/lateral orbitofrontal cortices compared with the healthy controls. In addition, the thalamo-SMC WM connectivity in patients with MCI was positively correlated with K-MMSE scores, and the thalamo-LTC connectivity was negatively correlated with ADAS-cog scores. This suggests that decreased thalamo-LPFC WM connectivity and reduced thickness of medial/lateral orbitofrontal cortices are closely related to MCI and/or early stages of AD.

It is widely recognized that WM abnormalities are detectable in the early stages of AD and MCI, suggesting that abnormalities in the cortico-cortical and cortico-subcortical WM interconnections are associated with an increased risk of progression from MCI to AD (Radanovic et al., 2013). A previous study (Wang et al., 2012) using VBM has reported that the MCI group showed lower fractional anisotropy (FA) and higher radial diffusivity (RD) in the parahippocampal WM compared with the control group. Another similar study (Palesi et al., 2012) demonstrated that patients with MCI showed decreased volume in the hippocampus and increased MD in the hippocampus-precuneus/posterior cingulate cortex tracts when compared with the healthy controls. The current study revealed that in comparison with the control group, the MCI group showed a significant decrease in the thalamo-LPFC WM connectivity. In addition, the thalamo-SMC WM connectivity in the MCI group was positively correlated with K-MMSE scores. The LPFC was shown to play an important role in cognitive function. There is evidence that cognitive deficits in patients with MCI are related to dysfunction of the LPFC (Duarte et al., 2006; Zhou et al., 2013). According to the study by Duarte et al. (Duarte et al., 2006), patients with MCI showed GM volume loss in the LPFC compared with the healthy controls. Zhou et al. (2013) evaluated the functional connectivity in the thalmo-cortical network in patients with AD. They reported a decrease in the functional connectivity between the left thalamus and left inferior frontal cortex. A cerebral perfusion study (Chao et al., 2009) demonstrated that MCI patients with executive dysfunctions showed hypoperfusion in the prefrontal cortex relative to controls. This indicates that thalamo-LPFC WM connectivity in the MCI patients may be associated with characteristics of the pathobiology of MCI.

We also found evidence that the decreased thickness of the medial/lateral orbitofrontal cortices in patients with MCI compared with healthy controls. The orbitofrontal cortex occupies the anterior part of the prefrontal cortex, which plays in complex human behaviors such as evaluation, affect regulation, and reward-based decision-making (Fettes et al., 2017). The orbitofrontal cortex atrophy was associated with cognitive deficits (Hornberger et al., 2010; Zhao et al., 2015). Both MCI and AD subjects had a thinner cortex in the lateral orbitofrontal cortex compared with healthy controls (Zhao et al., 2015). A positron emission tomography study (Mentis et al., 1995) demonstrated hypometabolism in the orbitofrontal cortex in patients with AD. Notably, observations with simultaneously decreased thalamo-LPFC WM connectivity, thickness in the orbitofrontal cortex, and reduced K-MMSE scores can be attributed to the cognitive dysfunction in the MCI patients.

Furthermore, atrophy in the temporal lobe and hippocampus has proven to be an important biomarker in the diagnosis of MCI and AD. Especially, hippocampal atrophy has also been an important predictor of progression from MCI to AD and may be a marker for early AD in patients with MCI (Jack et al., 1999, 2005; Apostolova et al., 2006; He et al., 2009). According to He et al. (2009), patients with amnestic multiple-domain MCI had significant hippocampal atrophy compared with the patients with amnestic single-domain MCI. The work of Apostolova et al. (2006) suggested that smaller hippocampal volume is associated with increased risk for conversion from MCI to AD. The current study found that patients with MCI showed significantly decreased volume in the hippocampus and middle temporal cortex compared with the healthy controls. This result is in concurrence with the finding that the most prominent structural changes at the initial stage in AD occur in the temporal lobe and hippocampus (Jack et al., 1999, 2005; Apostolova et al., 2006; He et al., 2009). Although the patients with MCI showed decreased GM volume in the middle temporal cortex, the significance level by multiple comparison correction is not high enough to validate this finding. In addition, the thalamo-LTC WM connectivity in the patients with MCI was negatively correlated with the ADAS-cog scores. The ADAS-Cog is a validated and robust scale for measuring the change in AD and continues to remain the regulatory standard outcome for AD trials (Schrag et al., 2012). Based on these findings, decreased thalamo-LPFC WM connectivity, reduced orbitofrontal cortical thickness, and hippocampal atrophy can be useful for diagnosing and tracking MCI.

In the current study, the MMSE scores in patients with MCI improved after donepezil treatment by 7.9%. Additionally, after 6 months of treatment, patients with MCI showed increased thalamo-MTC WM connectivity and higher GM volume in the inferior frontal cortex. However, the patterns of WM connectivity and brain volume change in untreated and treated patients were not significantly different from each other, resulting from multiple comparison corrections. However, these findings might not be clear due to the short follow-up period for donepezil treatment. Therefore, future studies comprising a larger population with a long period of follow-up are needed to generate more accurate and reliable data.

This study has some limitations. The small sample size means it does not have high statistical power. To compensate for this limitation, we considered a statistical threshold of *P*-value < 0.05 using FWE or Bonferroni correction. Another limitation relates to the quality inspection of DWI data, which is based on a visual inspection and individual processing in FSL software, without correction for the geometric distortion induced by B0 inhomogeneity. Thus, further correction through unwarping is needed to rectify susceptibility-induced geometric distortions. Another limitation involves the short follow-up period of about 6 months for donepezil treatment, hindering optimal evaluation of the time course of donepezil treatment efficacy. Therefore, large population studies with long-term follow-up are needed to obtain more accurate and reliable findings. Furthermore, a placebo-controlled study of MCI patients is recommended to validate and establish the efficacy of drug treatments.

## Conclusion

This study has demonstrated altered thalamo-cortical WM connectivity, cortical thickness, and GM volume following donepezil treatment in patients with MCI. More specifically, patients with MCI showed decreased thalamo-LPC WM connectivity and reduced thickness in the medial/lateral orbitofrontal cortices. Taken altogether, these findings could be valuable in the early detection of MCI as a prodromal phase of AD, as well as furthering understanding of the neurophysiopathological mechanism of MCI in connection with structural and functional abnormalities.

## Data Availability Statement

The data that support the findings of this study are available from the corresponding author upon reasonable request.

## Ethics Statement

The studies involving human participants were reviewed and approved by the Institutional Review Board of Chonnam National University Hospital. The patients/participants provided their written informed consent to participate in this study.

## Author Contributions

G-WK, S-EP, KP, and G-WJ designed the study, contributed to the analysis and interpretation of results, wrote the first draft of the manuscript. G-WK and G-WJ performed the majority of experiments. G-WJ has approved the final manuscript and completed manuscript. All authors agree with the content of the manuscript.

## Conflict of Interest

The authors declare that the research was conducted in the absence of any commercial or financial relationships that could be construed as a potential conflict of interest.
